# Lone Giant Atrium as a Variant of Atrial Cardiomyopathy: A Cardiovascular Magnetic Resonance Imaging Case Series

**DOI:** 10.3390/jcdd11100297

**Published:** 2024-09-24

**Authors:** Claudia Meier, Gabriel Olteanu, Marc Ellermeier, Michel Eisenblätter, Stephan Gielen

**Affiliations:** 1 Universitätsklinik für Kardiologie, Angiologie und Internistische Intensivmedizin, Universitätsklinikum Ostwestfalen-Lippe, Campus Klinikum Lippe, 32756 Detmold, Germany; claudia.meier@klinikum-lippe.de; 2 Department of Cardiology I, Division of Cardiovascular Imaging, University Hospital Münster, 48149 Münster, Germany; 3 Medizinische Fakultät, Universität Bielefeld, 33615 Bielefeld, Germany; michel.eisenblaetter@klinikum-lippe.de; 4 Universitätsinstitut für Diagnostische und Interventionelle Radiologie, Universitätsklinikum Ostwestfalen-Lippe, Campus Klinikum Lippe, 32756 Detmold, Germany; gabriel.olteanu@klinikum-lippe.de (G.O.); marc.ellermeier@klinikum-lippe.de (M.E.)

**Keywords:** giant atrium, echocardiography, cardiovascular magnetic resonance, reference values, rare diagnoses, atrial cardiomyopathy

## Abstract

Advances in cardiovascular imaging have expanded the scope and precision of rare diagnoses. Handling a patient with a giant left atrium, we focused on the existence and associated factors of “lone giant (left or right) atria” in our clinical setting. The aim of the current study was to establish reasonable cut-off values for the diagnosis of “giant atrium”. Our analysis utilised echocardiography and cardiovascular magnetic resonance (CMR) imaging databases, with the original data re-assessed to ensure consistency and comparability. Four patients met the search criteria, with two cases requiring CMR to confirm the diagnosis of “giant atrium”, correcting the initial echocardiographic assessment. Both echocardiography and CMR excel in the assessment of atrial anatomy, although the superior image quality and multiplanar capabilities of CMR support its preference. In assessing the atrial size, the use of 3D volumetric measurements should replace traditional biplane methods due to the complex anatomy of the atrium. We propose the use of an indexed volume threshold (>120 mL/m^2^) rather than simple diameter measurements for the diagnosis of “giant atria”. Structural atrial abnormalities appear to correlate with an increased risk of atrial arrhythmias, while potential serious complications such as thromboembolism or compression symptoms require further observation in larger patient cohorts to establish definitive risks.

## 1. Introduction

The term “giant left atrium” has been used in the literature to describe excessive left atrial dilatation and has never been clearly defined as a separate diagnosis. In 1956, Kent et al. first used the term to describe a series of 14 out of 153 patients who were assessed in the cardiac catheterisation laboratory for mitral valve disease [1]. The main indication for surgical therapy was dysphagia, first reported by Dines in 1966 [2] and termed “dysphagia megalatriensis” by Le Roux in 1969 [3,4]. Later, compressions of other intrathoracic neighbouring organs (left bronchus or left lung) were described [5].

Today, cardiac imaging by transthoracic echocardiography, computed tomography (CT), and cardiovascular magnetic resonance imaging (CMR) permits exact three-dimensional quantification of atrial dilatation. LA enlargement is defined as an LA maximal volume index (LAVi) higher than 34 mL/m2 or anteriorposterior diameter larger than 4 cm, measured by echocardiography, while definitions of giant left atrium varied from 6 cm to over 10 cm in diameter [3,5,6]. Hence, there is an increasing need to define cut-off values for the diagnosis of a giant atrium to find a common basis for communication, gathering and sharing experiences, and drawing therapeutic conclusions.

### 1.1. Left Atrium

In most publications, the term “giant atrium” is used indiscriminately for both primary (idiopathic) and secondary (left) atrial dilatation, which is disproportionate in relation to other accompanying cardiac pathologies such as left ventricular dysfunction or mitral valve disease. Today, there is a consensus that a giant atrium is not the result of pressure or volume overload alone, as it has also been observed in adults and children with normal mitral valve function [6] and may be related to atrial cardiomyopathies, which are not yet well classified [7]. Therefore, the diagnosis of “giant left atrium” should be reserved for primary atrial dilatation unrelated to severe mitral valve disease or left ventricular dysfunction. 

### 1.2. Right Atrium

Idiopathic right atrial enlargement is a rare abnormality observed in a wide age range with an unknown cause. Diagnosis involves ruling out other potential causes of secondary right atrial enlargement, such as pulmonary hypertension, massive pulmonary embolism, severe mitral valve disease, tricuspid stenosis, and Ebstein’s anomaly [8].

### 1.3. Modality-Dependent Approaches of Atrial Volumetry

The European Association for Cardiovascular Imaging (EACVI) recommends the use of biplane methods to assess LA volume because of limited access and experience with 3D volume measurements. Volumes derived from biplane methods that do not capture the entire dimension may be prone to measurement errors. These calculations rely on left ventricular long-axis views from both two- and four-chamber perspectives, which are generally about 60 degrees apart. Standard CMR long-axis images are aligned with the LV long axis, which often does not align with the true LA axis. In echocardiography, atrial-focused apical views are recommended for calculating LA volumes. Short-axis contouring in CMR has been suggested as a standard approach for validating LA volumes, yet its adoption in routine CMR practice has been hindered by the time required for scan acquisition and post-processing.

## 2. Methods

We retrospectively searched our imaging databases at the University Hospital OWL, Campus Klinikum Lippe, and the University Hospital Münster for patients with the diagnosis of “lone (right or left) giant atrium” within the last 3.5 years (1 January 2020–30 June 2024). The imaging databases included echocardiography and cardiovascular magnetic imaging. The original data were re-evaluated and analysed for consistent and comparable values. Endsystolic biplane diameter, biplane area, and the total volume were analysed and indexed to body surface area, excluding the supplying veins and appendages. For echocardiography, Philips IntelliSpace Cardiovascular©, and for CMR-data Circle CVI 24 Cardiovascular Imaging© software package was used for data analysis. At the time of imaging data collection, patients had previously consented to subsequent anonymised data analysis for scientific purposes.

To establish a reproducible and comprehensible cut-off value for the diagnosis of “giant atrium”, we suggest twice the maximal normal value of atria + 20% (resulting in > 120 mL/m^2^) and check whether our patients fulfilled this criterion.

## 3. Results

**Figure 1:**
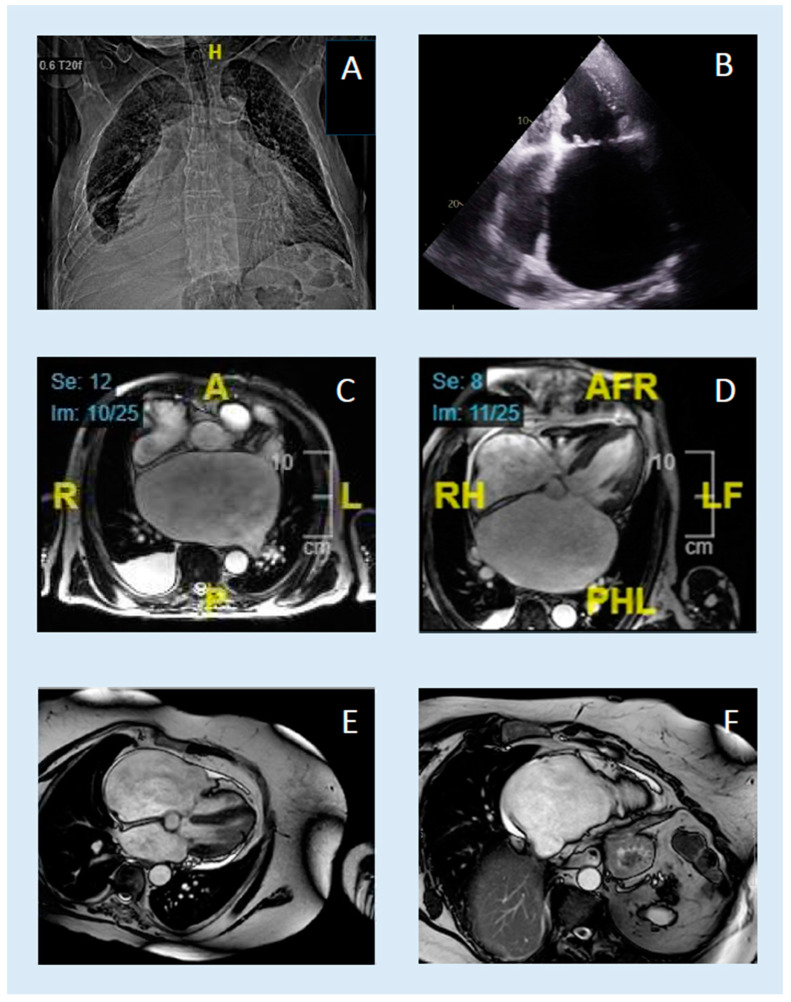
Examples of different imaging methods in “giant atrium“. (**A**–**D**): Patient A with *left giant atrium.* (**E**,**F**): Patient B with *right giant atrium.* A: Chest X-ray ap; B: Thansthoracic echocardiography apical 4-chamber view in endsystole; C: transversal bSSFP-cine CMR in endsystole; D: 4-chamber bSSFP-cine CMR in endsystole; E: 4-chamber bSSFP-cine CMR in endsystole; F: right focussed 2-chamber bSSFP-cine CMR in endsystole. H: head; F: feet; A: anterior; P: posterior; R: right; L: left.

In summary, four patients fulfilled the search criteria mentioned above. One patient with giant left atrium (patient A) was initially diagnosed by echocardiography and the remaining three patients (patients B–D) with giant right atrium by CMR. In each case, this was the time of the initial diagnosis. Details of the clinical circumstances and the respective measurement data can be found in Table 1 and Table 2. Exemplary images from MRI and echocardiography are provided in Figure 1. Since the right atrial enlargements were all found by CMR, it was confirmed in our case series that CMR is the superior imaging method to detect right atrial enlargements.

All patients fulfilled the predefined criterion for a “giant atrium” with a size > 120 mL/m^2^.

## 4. Discussion

When considering cardiac normal values, one is initially faced with the problem of inconsistent definitions, as different subjects with different imaging procedures have different reference values.

The assessment of the cardiac dimensions is usually made by ultrasound, as a chest X-ray is indicative but not accurate enough. Several studies and societies have published echocardiographic reference values, for example, the European Association of Cardiovascular Imaging (EACVI) and the American Society of Echocardiography (ASE) [9], the NORRE (Normal Reference Ranges for Echocardiography) study [10], or the EchoNORMAL (Echocardiographic Normal Ranges Meta-Analysis of the Left Heart) study [11]. According to the recommendations of the American and European Societies of Echocardiography, LA enlargement is defined as an LA maximal volume index (LAVi) higher than 34 mL/m^2^. A recent study suggested new left atrial maximum volumes of 44-53 mL/m^2^, which is above the previously published references [12].

CMR can serve as an alternative imaging method. For left atrial functional analysis, MRI is regarded as the gold standard technique, addressing many of the inherent limitations of echocardiographic assessment [13]. The literature lacks consensus on the best method to measure left atrial volume in CMR. The most common methods are the modified Simpson’s method in short axis views and the biplane area-length method on 2- and 4-chamber views on cine bSSFP images, excluding the pulmonary veins and appendage during ventricular systole before the mitral valve opens. One group showed that LA volumes obtained with the LA-focused long-axis biplane method did not differ significantly from the reference method of short-axis stack volumetry without much effort and time delay [14]. Indexed reference values for left atrium volume are 40 +/− 8 mL/m^2^ and 52 +/− 12 mL/m^2^ for the right atrium in a pooled meta-analysis for CMR [15]. At this point, it should only be mentioned that body surface area, age, sex, and ethnicity have been shown to influence atrial size [12,16].

Definitions of a giant left atrium have varied, with some ranging from 6 cm to over 10 cm in diameter. One definition of the criteria includes (a) a large left atrium on M-mode echocardiography with a diameter greater than 65 mm and (b) the left ventricular postero-basal wall curving inward and positioned between the dilated left atrial cavity and the left ventricular cavity [6]. 

In our patients, it was interesting that the absolute and indexed volumes on the CMR images did not always match the subjective impression of size in the 4- and 2-chamber view. Therefore, 3D volume measurements (measured with end-systolic volumetry on axial cine stacks) should be preferred over simple biplane determination of size, as the atrial anatomy is too complex for a single 2D representation. The approach should be simple and reproducible, so an indexed atrial volume based on body surface area seems suitable. Since the normal indexed reference values for both atria vary around the maximal 50 mL/m^2^, we suggest twice the maximal normal value + 20% (resulting in > 120 mL/m^2^) for diagnosing a “giant atrium”.

To support this thesis and to receive an impression of how large “diseased atria” are on average, irrespective of atrial fibrillation, we looked at the following study as an example because secondary left atrium cavity remodelling is common in heart failure patients: Rossi et al. measured left atrial size by echocardiography in a large cohort of heart failure patients without atrial fibrillation. The worst group with an enlarged left atrium and abnormal function showed an indexed LA size (LAVi) of 57 ± 16 mL/m^2^ [17]. If the fourth standard deviation is calculated on the average value, a size of 121 mL/m^2^ is obtained, which is to be regarded as disproportional even for secondary diseased atria. This value corresponds very well with our proposal. All our patients meet this cut-off criterion using this simple method.

The cause of giant atria seems to be congenital or acquired, and congenital enlargement results not only from pressure overload but also from weakness of the atrial wall. They are associated with young age, have no associated valvular abnormality, and may be undetected because of often missing symptoms. Aneurysm resection and left atrial reconstruction are the recommended treatments [10]. In contrast, the acquired giant atria can be traced back to a pressure or volume overload. It is often seen in the setting of rheumatic heart disease, becoming less common as the incidence of rheumatic fever decreases. Symptoms can be explained by displacement of mediastinal structures, e.g., shortness of breath due to displacement of lung volume or atrial arrhythmias [11]. This is another advantage of MRI, as not only the heart but also the surrounding medical structures can be assessed very well. In our case, the displacement of the lung tissue in patient A is particularly easy to see. This plays an important role in assessing the indication for surgery.

Our cohort is too small to draw statistically valid conclusions about aetiology or prognosis, but an accumulation of symptoms and associated conditions can be observed. Due to the disruption of the atrial structure, there seems to be a tendency towards atrial arrhythmias. However, it cannot be definitively stated whether the structural changes or the arrhythmia occurred first, as both pathologies can mutually exacerbate each other. Nevertheless, since the vast majority of patients with atrial tachycardia do not have a “giant atrium” despite a certain enlargement, this appears to be an independent atrial cardiomyopathy. Additionally, there are cofactors that promote atrial dilation, such as a patent foramen ovale or atrioventricular valve insufficiencies. However, since these were not assessed as severe and are common in the general patient population, they cannot be solely considered as the explanation for a “giant atrium”. The age of the patients was 44–89 years at first diagnosis, with the oldest patient suspected of having atrial abnormalities since adolescence. This suggests that at least this patient may have a congenital form of giant atrium. 

## 5. Conclusions

In summary, rare phenomena, such as “lone giant atria”, are observed more frequently with improved technology and more frequent use of multimodal imaging. Echocardiography and CMR are well suited for anatomical assessment of the atria, although CMR should be preferred due to overarching image quality and free choice of axis. We strongly recommend the assessment of the indexed 3D volume over biplane 2D views to capture the full size of complex anatomical structures and not be influenced by subjective “eyeballing” impressions. Therefore, we suggest not using the previous approach of biplane diameters for diagnosing a giant atrium but instead an indexed volume of > 120 mL/m^2^. 

## Figures and Tables

**Table 1:** Patients characteristics.

Patient	Age at First Diagnosis	Sex	BSA (m²)	Symptoms	diagnosis Prior to Imaging	Diagnosis After Imaging	Atrial Arrhythmia	Therapie	Follow-Up (in Month Sice Diagnosis)	Associated Malformations	Imaging Method for Data Analysis
A	89 (Anomaly suspected by chest X-ray since adolescence)	male	2.0	dyspnoea, dizziness, syncope	Cardial decompensation with reduced LV function	no reduced LV function, giant **left** atrium, moderate mitral regurgitation	permanent atrial fibrillation	recompensation with diuretics	No symptoms (5)	none	2D-Echo + CMR
B	66	female	2.2	stroke	Ebstein’s anomaly	no Ebstein, giant **right** atrium	none	anticoagulation	Implantation of PFO-occluder because of stroke (49)	PFO (not haemodynamically relevant)	CMR
C	53	male	2.4	tachykardia	Uhl´s disease	no Uhl, giant **right** atrium	atrial tachycardia	antiarrhythmic medication	No symptoms (30)	none	CMR
D	44	female	1.7	tachykardia	Tricuspid valve prolapse	mild prolapse with mild tricuspid regurgitation, giant **right** atrium	atrial tachycardia	ablation therapy	Ablation of 2 atrial tachycardias in the CS ostium (22)	tricuspid prolapse, PFO (not haemodynamically relevant)	CMR

**Table 2:** Imaging parameters.

Patient	Imaging Method for Data Analysis	LVEF (%)	LVEDV (mL)	LA Volume Endsystolic, Measured by Modified Simpson’s Method in Atrial Short Axis Views	LA Volume Indexed (mL/m²)	Diameter Apical 4Ch View Endsystolic (mm)	Diameter Apical 2Ch View Endsystolic (mm)	LA Area Apical 4Ch View Endsystolic (cm²)	LA Area Apical 2Ch View Endsystolic (cm²)	“Eyeballing” Impression Only Based on 2D Biplane Images
A	CMR	63	184	1472	747	131 × 134	132 × 145	151	138	Diagnosis confirmed
				**RA Volume Endsystolic (cm³ = mL), Method See Above**	**RA Volume Indexed (mL/m²)**	**Diameter 4Ch View Endsystolic (mm)**	**Diameter RV-2Ch View Endsystolic (mm)**	**RA Area 4Ch View Endsystolic (cm²)**	**RA Area RV-2Ch View Endsystolic (cm²)**	
B	CMR	67	122	338	154	64 × 112	86 × 116	59	75	Diagnosis confirmed
C	CMR	63	219	551	220	84 × 95	95 × 98	70	78	Diagnosis considered
D	CMR	62	145	214	126	52 × 59	not available	29	not available	Diagnosis not expected

## Data Availability

The data that support the findings of this study are not openly available due to reasons of sensitivity and are available from the corresponding author upon reasonable request.
